# Supplementary figures and images for: Computational investigation of missense somatic mutations in cancer and potential links to pH-dependence and proteostasis
Source: PLoS One. 2024 Nov 19;19(11):e0314022. doi: 10.1371/journal.pone.0314022 (PMC11575792; doi:10.1371/journal.pone.0314022)

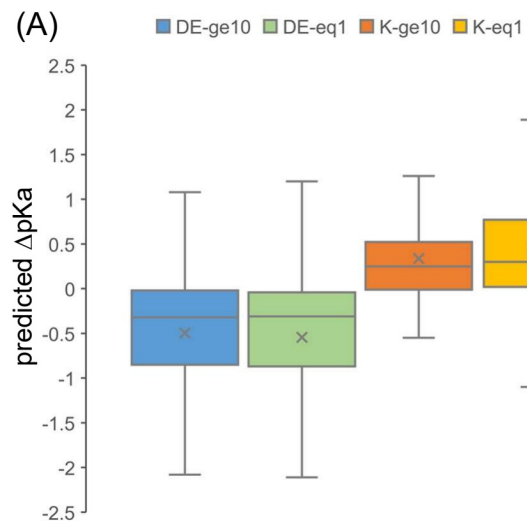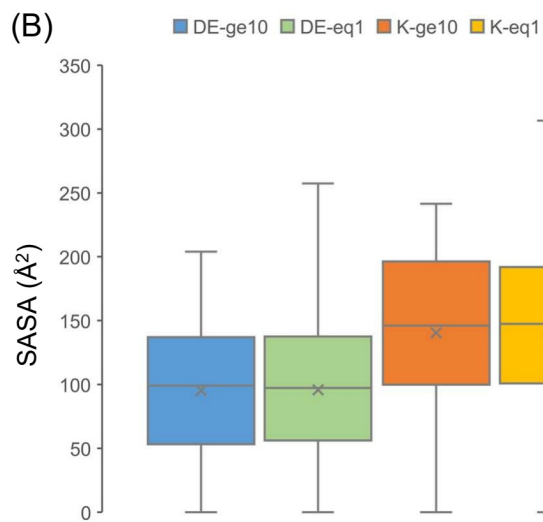

Supplement: S1 Fig — The box and whisker plots show quartiles, median (central line), and mean (cross). (A) Distributions of predicted Asp (D), Glu (E), and Lys (K) ΔpKas (pkcalc) are shown for instEQ1 and instGE10 mutation data. Limiting thresholds of +/- 3 are applied for ΔpKa values. (B) The ΔpKa data for mutations in panel (A) are replaced with SASA, for the same subsets. (PDF) [file pone.0314022.s001.pdf]
