# Supplementary material for: Computational investigation of missense somatic mutations in cancer and potential links to pH-dependence and proteostasis
Source: PLoS One. 2024 Nov 19;19(11):e0314022. doi: 10.1371/journal.pone.0314022 (PMC11575792; doi:10.1371/journal.pone.0314022)
Supplement: Attachment — Submitted filename: PONE-D-24-29679_review_report.pdf [file pone.0314022.s002.pdf]

Review Report:

The paper explores the role of somatic mutations, with a particular focus on arginine depletion and pH-sensing mutations, in cancer cell adaptation to acidic environments and their potential impact on tumor progression. The study employs advanced computational tools, including AlphaFold2 and pKa prediction methods, to analyze the structural and functional implications of these somatic mutations.

The paper highlights potential therapeutic opportunities by targeting pH-sensing mechanisms in cancer cells.

Major Comments:

1. The paper primarily focuses on histidine and arginine mutations, neglecting other potentially relevant mutations that could provide a more comprehensive understanding of cancer cell adaptation. Expanding the analysis to include other amino acid mutations that may play a role in pH-dependent processes would offer a more holistic understanding of the mechanisms involved.

2. The introduction and background sections do not provide enough context on the broader landscape of somatic mutations in cancer, limiting the reader's understanding of the study's significance. Enhancing these sections to include a more detailed discussion of the general landscape of somatic mutations in cancer will help readers better appreciate the study's relevance and significance.

3. The paper places too much emphasis on computational data without adequately discussing the limitations and potential inaccuracies of these methods. A more balanced discussion is needed, critically evaluating the limitations of the computational methods used and discussing potential inaccuracies and their impact on the study's conclusions.

Minor Comments:

1. The introduction should be expanded to provide more context on the broader landscape of somatic mutations in cancer, which is crucial for understanding the study's significance.

2. The results are presented clearly, but the discussion does not adequately address the computational methods' limitations. Additionally, the focus on histidine and arginine mutations is too narrow.

Conclusion:

The paper provides valuable insights into the role of arginine depletion and pH-sensing mutations in cancer. However, major revisions are required to address the lack of experimental validation, narrow focus, and insufficient contextual background. With these revisions, the study could make a substantial contribution to the field.
