# Supplementary material for: Computational investigation of missense somatic mutations in cancer and potential links to pH-dependence and proteostasis
Source: PLoS One. 2024 Nov 19;19(11):e0314022. doi: 10.1371/journal.pone.0314022 (PMC11575792; doi:10.1371/journal.pone.0314022)
Supplement: Attachment — Submitted filename: Reviewer comments.docx [file pone.0314022.s003.docx]

Comments to the Authors:

The study titled "Computational Investigation of Missense Somatic Mutations in Cancer and Potential Links to pH-Dependence and Proteostasis" by Shalaw Sallah and Jim Warwicker provides a thorough analysis of somatic missense mutations, specifically focusing on the arginine-to-histidine substitution, which may contribute to pH-sensing functions. Within the frequently mutated subset, the authors identified mutations in NDST1, the HLA-C chain of the MHC I complex, and the water channel AQP-7 as potential mediators of pH-dependence in cancer cells. Furthermore, they emphasized that rebalancing the arginine-to-lysine ratio is crucial for maintaining proteostasis in peripheral cellular locations and controlling tumor development. However, I believe there are areas where further improvements could be made.

Major revisions:

1. In the methods section, the authors noted their use of COSMIC database version 97. However, the latest version, 100, has been released, including several new missense mutations. Did the authors consider analyzing these recently added mutations in the current study? If not, it would be beneficial to include this analysis.
2. In Table 1, ten mutations to histidine are listed along with mutation specificity and pKa values. However, the parameters used to analyze the stability of the protein structures are not clearly defined. Please provide the exact SASA (Solvent Accessible Surface Area) values for each mutation. Additionally, include the SIFT, PolyPhen, and CADD (Combined Annotation Dependent Depletion) scores for each mutation to enhance clarity.
3. In Section 3.7, the authors described how the lysine-to-arginine balance could be beneficial for maintaining cellular proteostasis in tumor cells. Have the authors analyzed Lysine/Arginine (Lys/Arg) mutations in previously published studies on centenarians or healthy aging? Does this Lys/Arg balance contribute to healthy aging, potentially acting oppositely to the mechanism observed in cancer cells?

Minor Revisions:

4. The authors discussed the significance of the arginine-to-histidine mutation in cancer cells, highlighting its role in gaining pH-sensing function. They also referenced previously published studies explaining the mechanism behind this mutation. However, are there any other significant functions gained by this arginine-to-histidine mutation specifically in cancer cells beyond pH-sensing? If so, please elaborate on these in the introduction.
5. In Section 3.4.2, the authors discussed the stabilization of the protein structure. Were any specific parameters used to measure this stabilization in the predictive analysis? Please add the SASA values or other relevant measures in brackets next to the sentences where stabilization is explained.
6. In Section 3.9, the authors explained that genes with subsets of mutations from arginine to lysine were analyzed using Gene Ontology (GO) pathways, and these mutations were found to be enriched in the "cell periphery" GO component category. However, the methodology for this analysis is unclear. The authors should provide more details on how the "cell periphery" pathway was identified. Was the most significant pathway selected?
7. In the conclusion, on line 623, the authors cited a paper suggesting that Arg/Lys rebalancing could be tested with wild-type and arginine-depleted membrane proteins in a cell-based assay. Please provide more experimental results supporting the role of the Arg/Lys rebalancing mechanism in maintaining proteostasis in cancer cells, along with relevant citations.

Dear Editor,

Thank you for the opportunity to review the manuscript titled "Computational Investigation of Missense Somatic Mutations in Cancer and Potential Links to pH-Dependence and Proteostasis" by Shalaw Sallah and Jim Warwicker. After thoroughly evaluating the study, I would like to provide my feedback and recommendations.

Summary:

The authors have conducted analysis of somatic missense mutations, specifically focusing on the arginine-to-histidine substitution, which may contribute to pH-sensing functions. Within the frequently mutated subset, the authors identified mutations in NDST1, the HLA-C chain of the MHC I complex, and the water channel AQP-7 as potential mediators of pH-dependence in cancer cells. Furthermore, they emphasized that rebalancing the arginine-to-lysine ratio is crucial for maintaining proteostasis in peripheral cellular locations and controlling tumor development. Overall, the study is well-structured and contributes significantly to the field.

**Areas for Improvement:**

Major revisions:

1. In the methods section, the authors noted their use of COSMIC database version 97. However, the latest version, 100, has been released, including several new missense mutations. Did the authors consider analyzing these recently added mutations in the current study? If not, it would be beneficial to include this analysis.
2. In Table 1, ten mutations to histidine are listed along with mutation specificity and pKa values. However, the parameters used to analyze the stability of the protein structures are not clearly defined. Please provide the exact SASA (Solvent Accessible Surface Area) values for each mutation. Additionally, include the SIFT, PolyPhen, and CADD (Combined Annotation Dependent Depletion) scores for each mutation to enhance clarity.
3. In Section 3.7, the authors described how the lysine-to-arginine balance could be beneficial for maintaining cellular proteostasis in tumor cells. Have the authors analyzed Lysine/Arginine (Lys/Arg) mutations in previously published studies on centenarians or healthy aging? Does this Lys/Arg balance contribute to healthy aging, potentially acting oppositely to the mechanism observed in cancer cells?

Minor Revisions:

4. The authors discussed the significance of the arginine-to-histidine mutation in cancer cells, highlighting its role in gaining pH-sensing function. They also referenced previously published studies explaining the mechanism behind this mutation. However, are there any other significant functions gained by this arginine-to-histidine mutation specifically in cancer cells beyond pH-sensing? If so, please elaborate on these in the introduction.
5. In Section 3.4.2, the authors discussed the stabilization of the protein structure. Were any specific parameters used to measure this stabilization in the predictive analysis? Please add the SASA values or other relevant measures in brackets next to the sentences where stabilization is explained.
6. In Section 3.9, the authors explained that genes with subsets of mutations from arginine to lysine were analyzed using Gene Ontology (GO) pathways, and these mutations were found to be enriched in the "cell periphery" GO component category. However, the methodology for this analysis is unclear. The authors should provide more details on how the "cell periphery" pathway was identified. Was the most significant pathway selected?
7. In the conclusion, on line 623, the authors cited a paper suggesting that Arg/Lys rebalancing could be tested with wild-type and arginine-depleted membrane proteins in a cell-based assay. Please provide more experimental results supporting the role of the Arg/Lys rebalancing mechanism in maintaining proteostasis in cancer cells, along with relevant citations.
